# Capillary Malformation-Arteriovenous Malformation Syndrome

**DOI:** 10.7759/cureus.12562

**Published:** 2021-01-07

**Authors:** Razan Alluhaibi, Layan N Alkhayat, Wajd Aqeeli

**Affiliations:** 1 Dermatology, King Abdulaziz Hospital, Makkah, SAU; 2 Dermatology, Umm Al Qura University, Makkah, SAU

**Keywords:** rhodoid nevus, capillary malformations (cms), capillary malformations-arteriovenous malformation (cm-avm), rasa1 gene, rasp21 protein

## Abstract

Capillary malformation-arteriovenous malformation (CM-AVM) is an autosomal dominant inherited rare type of vascular malformation encountered in a neonate and first described in 2003. It has been reported in association with heterozygous mutations in the RASA1 gene, which encodes the protein RASp21. In 2010, a German doctor proposed rhodoid nevus as a name for this type of capillary malformation; in ancient Greek, rhodoides means “rose-like” or “rose-colored.” Accordingly, CM-AVM could also be called “rhodoid nevus syndrome.” We report this case as its very challenging diagnosis with its further differentials and its association with thrombocytopenia.

## Introduction

The development of the dermal vessels requires penetration of capillary vessels, which is induced by vascular endothelial growth factor (VEGF) that is secreted by keratinocyte in the avascular epidermis. Any defect in this cutaneous vascular development reveals malformed vessels that vary in location, size, blood flow, and clinical severity. Although CMs are common, CM-AVM is a newly recognized autosomal dominant disorder that has been reported in association with heterozygous mutations in the RASA1 gene, which encodes the RASp21, a protein involved in growth factor signaling for cell proliferation, migration, and survival [1].The prevalence of CM-AVM syndrome is approximately one in 100 000, and in a study of 44 families, 32% of mutations were found to be de novo. The penetrance is between 89% and 98.5% [2]. CMs are flat, cutaneous, slow-flow lesions composed of dermal capillary-venular-like channels that are dilated and/or increased in number. Those CMs may be solitary in a small minority of cases, and some CMs have a small white halo, suggesting vascular steal (high flow with direct arteriovenous shunting). However, CMs may be the only finding in some affected individuals; arteriovenous malformation (AVM) and arteriovenous fistula (AVF) are fast-flow vascular anomalies that can arise in the skin, muscle, bone, internal organs, and the brain and can cause life-threatening complications such as bleeding, congestive heart failure, or neurologic consequences [1,3].

## Case presentation

**Figure 1:**
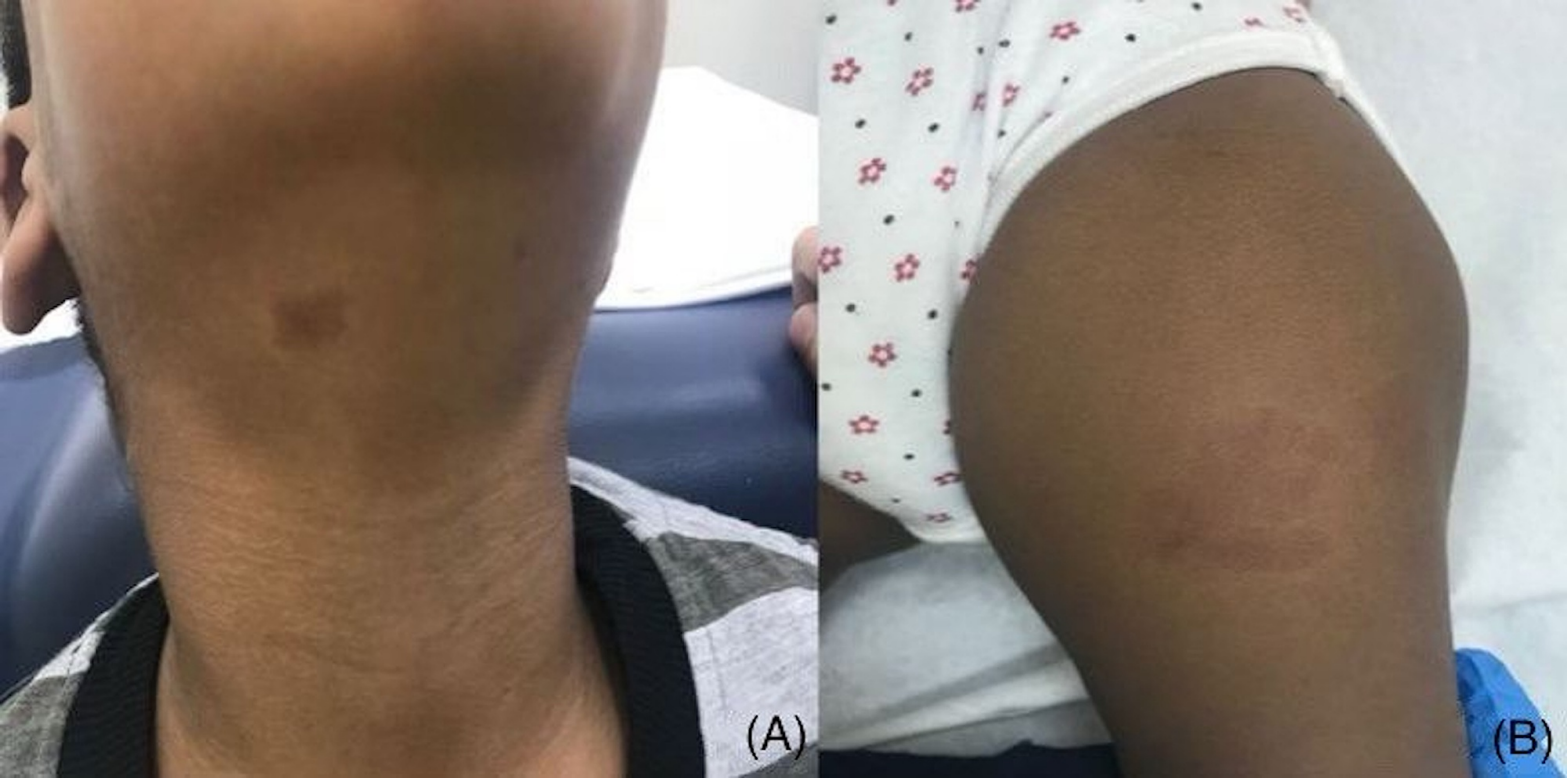
The patient's brother (A) and sister (B).

**Figure 2:**
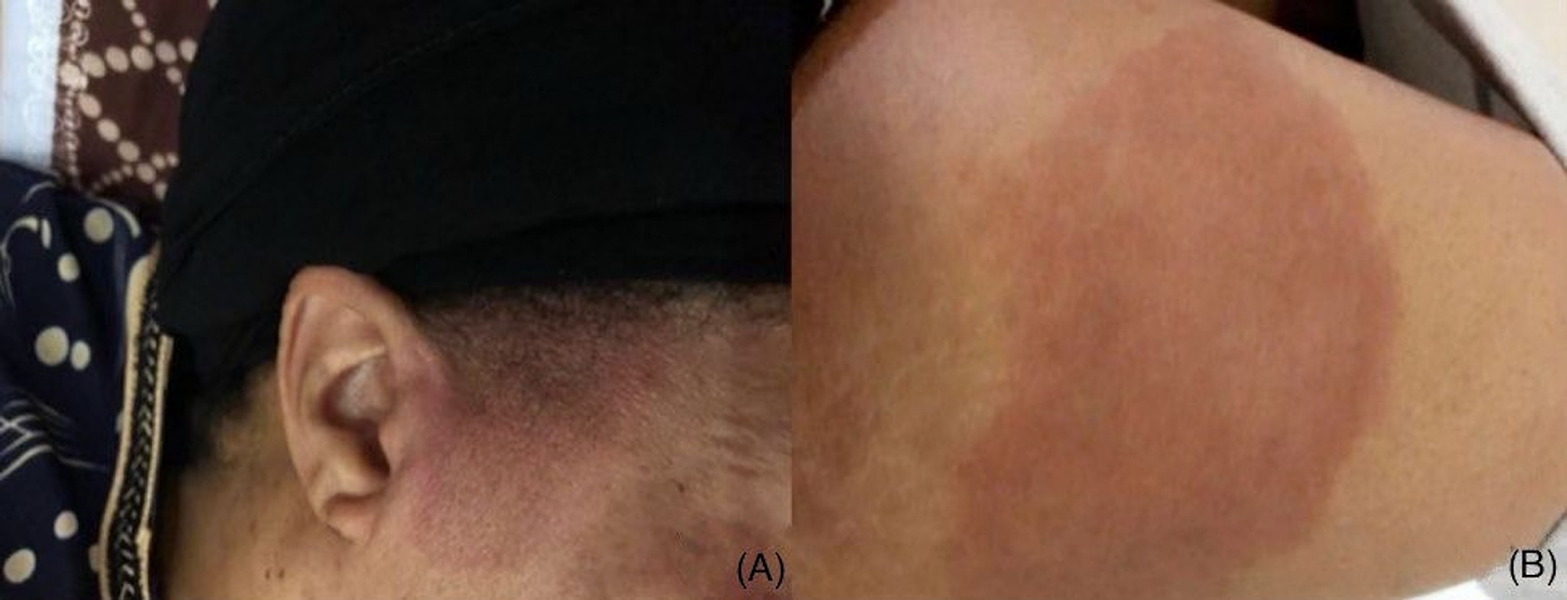
Patient's grandmother (A) and her aunt (B).

**Figure 3:**
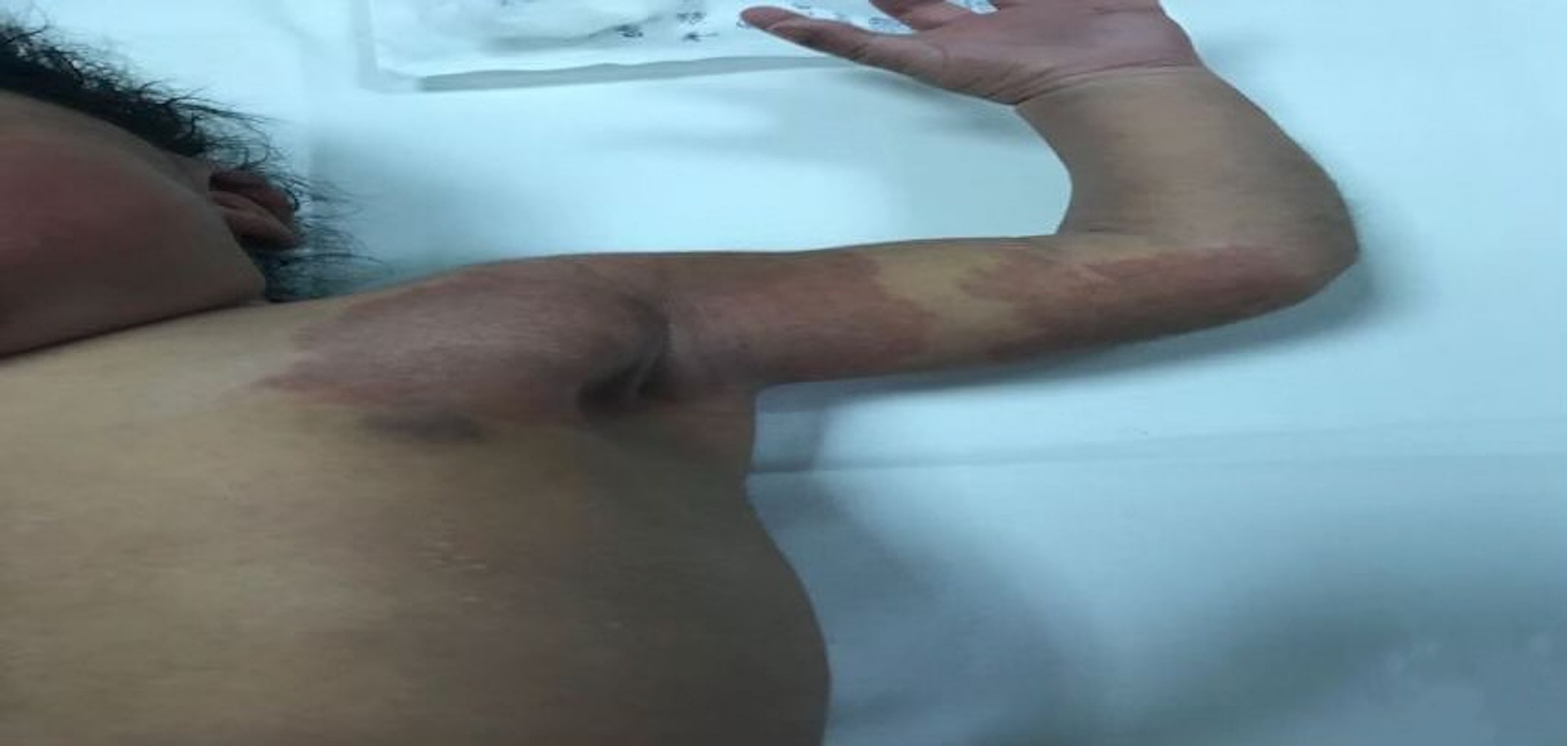
A well-defined erythematous patch over the left shoulder and arm measuring 4 cm x 3 cm with irregular borders.

**Figure 4:**
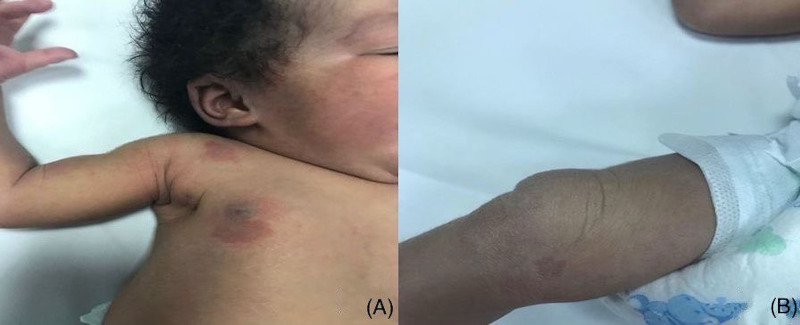
Multiple smaller round to oval erythematous macules and patches on the right side of the chest (A) and over the right knee (B).

A three-day old female patient in the nursery ward was consulted for assessment of multiple, red skin lesions on the chest and left shoulder. The baby was born full-term and by a spontaneous vaginal delivery. Soon after delivery the patient noticed to have low platelet count for which a pediatric hematologist was consulted, and the patient diagnosed with autoimmune thrombocytopenia, treated with platelet transfusion and intravenous immunoglobulin (IVIG). Two of the patient's siblings were also born with similar skin lesions and diagnosed with autoimmune thrombocytopenia (Figure 1). Her grandmother and two of the patient’s aunts had a history of similar lesions, which were stable since birth. On receiving the mother's history, all her previous pregnancies were complicated with gestational thrombocytopenia (Figure 2). The patient's parents showed positive consanguinity. All affected family members are completely normal with normal development/growth and no systemic symptoms. On examination, the patient was vitally stable with normal growth parameters, no syndromic features, macro/microcephaly, or limb deformities. The physical examination showed well-defined red to rose-colored patch over the left shoulder and arm measuring 4 cm x 3 cm with irregular borders (Figure 3); also, there were multiple smaller round to oval erythematous macules and patches on the right side of the chest and over the right knee (Figure 4). There was no tenderness or thrill. Nails, mucous membrane, and hair examination were unremarkable; the patient showed a normal systemic examination including eye and neurologic examinations. Histopathologic findings showed dilated capillary venular channels in the upper dermis and no arteriovenous malformations. Ultrasound was done for the patient, and the report revealed dilated dermal postcapillary venule with an increase in Doppler flow compared to the adjacent dermis. There were no fistula and no arteriovenous or lymphatic malformations. Genetic studies were not done due to the patient's family refusal. The patient was finally diagnosed with a distinct type of capillary malformation-arteriovenous malformation syndrome newly named as rhodoid nevus. The patient's parents were reassured and a pulse dye laser treatment was offered to be done in a later stage of the patient's life.

**Figure 5:**
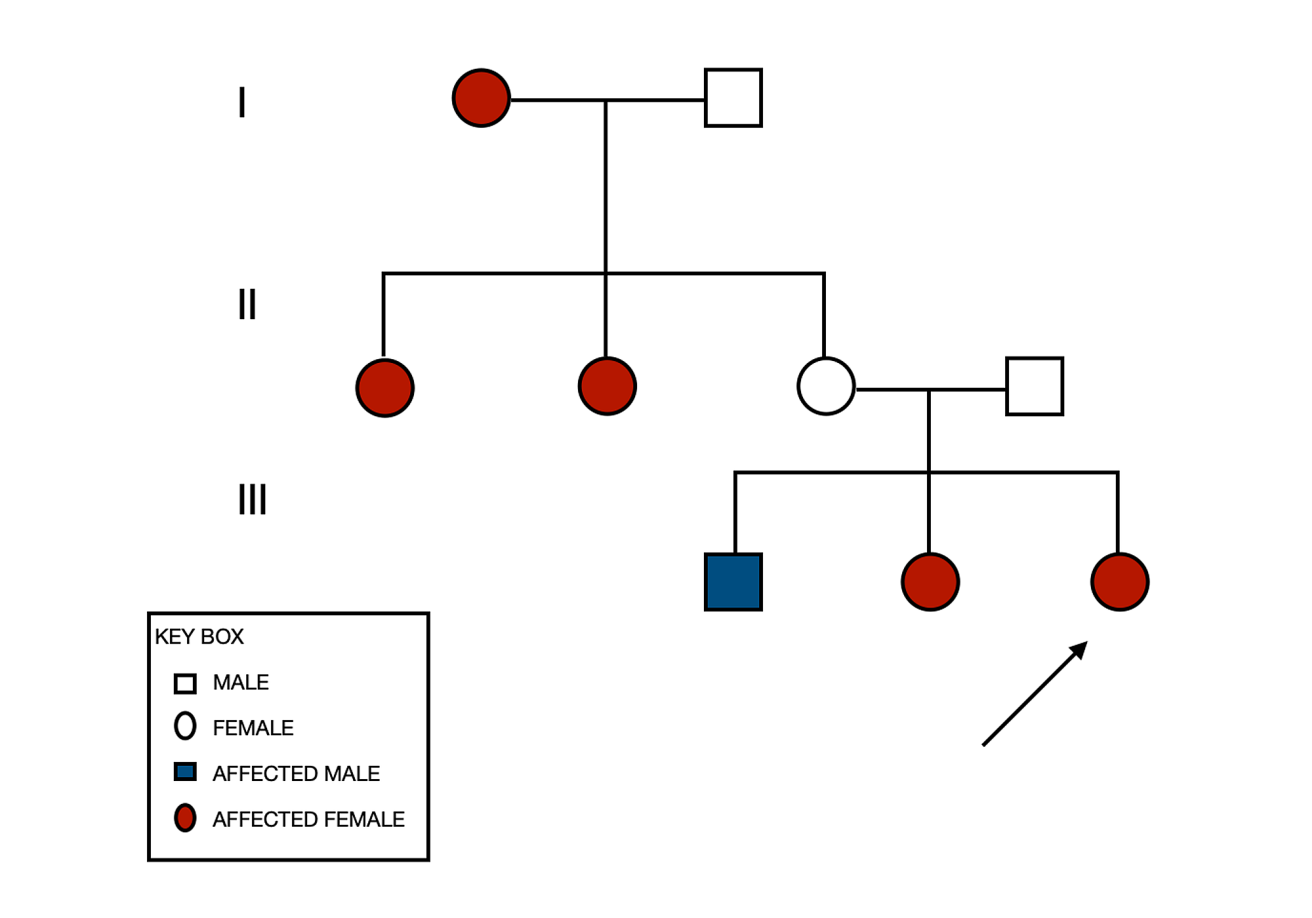
Pedigree chart of the autosomal dominant pattern of the disease through the family.

## Discussion

In 2003, a distinct autosomal dominant trait characterized by multiple, small capillary malformations of round or oval shape was described by Miikka Vikkula's team from Brussels. They found that this phenotype that had previously been mapped to chromosome 5q was caused by RASA1 mutations. Because the skin lesions were sometimes associated with a fast-flow arteriovenous malformation of the Parkes Weber type, they chose the name “capillary malformation-arteriovenous malformation (CM-AVM)” [4]. In 2010, a German university doctor named Rudolf Happle proposed a term of rhodoid nevus for this capillary malformation to distinguish it from other types of capillary malformations; its color is lighter than nevus flammeus, but when it comes to comparing it to nevus roseus, its tone is sometimes darker. Both nevus roseus and nevus flammeus are archetypically arranged in a checkboard pattern like segmental or flag-like and sometimes systemized. On the other hand, rhodoid nevus tends to be small, round, or oval and appears in haphazard distribution. Genetically rhodoid nevi are autosomal dominant, while both of nevus roseus and nevus flammeus mostly exist sporadically. The term “capillary malformation” includes additional several vascular lesions that present with nevi, e.g., angiokeratoma circumscriptum, cutis marmorata telangiectatica congenita, and nevus anemicus. On the other hand, vascular lesion does not present with nevi just as the salmon patch and the telangiectatic lesions of Rendu-Osler disease [1,5].

The capillary malformation of rhodoid nevus could be sometimes associated with arteriovenous malformation for which we can call it rhodoid nevus syndrome [1]. Regarding our case, the presence of thrombocytopenia in all the family members with the rhodiod nevus could be coincidental findings or associated with the rhodoid nevus, which needs further review. As there is no causal therapy, only cosmetic treatment of rhodoid nevi may be performed by pulse dye laser or photodynamic therapy. Likewise, it may include the arteriovenous malformations treatment, which depends on the location of the malformation. It may include embolization, surgery, and/or other special treatments [6,7].

## Conclusions

The rhodoid nevus, formerly CM-AVM, is a distinct AD trait characterized by multiple, small capillary malformations of round or oval shape, caused by RASA1 mutations, sometimes associated with a fast-flow AVM. Further investigations are warranted for the rhodiod nevus association with thrombocytopenia.
